# About the Acceptance of Wearing Face Masks in Times of a Pandemic

**DOI:** 10.1177/20416695211021114

**Published:** 2021-05-30

**Authors:** Claus-Christian Carbon

**Affiliations:** Department of General Psychology and Methodology, University of Bamberg, Germany

**Keywords:** perceived strangeness, social acceptance, COVID-19, virus, face masks, psychology, pandemic

## Abstract

Wearing face masks in times of COVID-19 is one of the essential keystones for effectively decreasing the rate of new infections and thus for mitigating the negative consequences for individuals as well as for society. Acceptance of wearing masks is still low in many countries, making it extremely difficult to keep the pandemic at bay. In an experimental study, participants (*N* = 88) had to assess how strange they felt when wearing a face mask while being exposed to displays of groups of varying numbers of mask wearers. Three different types of face masks were shown: simple homemade masks, FFP2 masks, and loop scarfs. The higher the frequency of people wearing masks in the displayed social group, the less strange the participants felt about themselves, an essential precondition for accepting wearing masks. This effect of a descriptive social norm was particularly effective when people saw others wearing less intrusive masks, here, simple homemade masks.

The World Health Organization Strategic and Technical Advisory Group for Infectious Hazards (STAG-IH) regularly reviews and adjusts the assessment of risks and needed measures to mitigate the infection of SARS-CoV-2 (severe acute respiratory syndrome coronavirus 2) causing COVID-19. One of the pragmatic ways of reducing the chance of transmitting respiratory viruses, in general, is to use face masks^1^ (Jefferson et al., 2008), which has recently been recommended for the specific situation of COVID-19 as well (Jefferson et al., 2020).

Besides providing a physical barrier to the virus, face masks can have further functions: They can, for instance, cue adequate hygienic behaviour in a social situation, they may trigger additional, positive hygiene practices (Wada et al., 2012), and they can reduce fears and thus facilitate active partaking in social life (Olivera-La Rosa et al., 2020), especially for very vulnerable persons or people with an intolerance of uncertainty (Taylor, 2019).

Wearing masks is not a sufficient (Mniszewski et al., 2014) but a necessary facet of the full spectrum of interventions set up to delay a major surge of the pandemic and to level the demand for hospital beds while protecting those persons who are most vulnerable to a severe case of COVID-19 (elderly people, people with respiratory problems and other comorbidities; Wu & McGoogan, 2020).

Although the multifaceted benefits of face masks are sufficiently known nowadays (Chu et al., 2020), the usage of masks was not unanimously seen positive. For instance, especially in the early times of COVID-19, actually, when the present study was conducted in April 2020, where everyday experiences were still rare, even official sites criticised the usage of face masks, for instance, because people might incorrectly use the masks (World Health Organization, 2020), increase hazardous hand–face contacts when using masks (but see Tao et al., 2020), or because masks might create a false sense of security yielding to reduced social distancing or other hygiene practices (World Health Organization, 2020). Particularly the wearing of professional masks (e.g., FFP2 or FFP3) in areas outside of the health sector was seen critically due to unsecured logistics, empty storages, and “unnecessary costs” (World Health Organization, 2020, p. 1). During the course of the pandemic, it turned out that massive mask usage causes significant pollution of beaches (Ardusso et al., 2021) and other public places (Kumar et al., 2020). People are hard to be recognised when wearing masks (Carragher & Hancock, 2020), emotional reading is substantially hampered, causing characteristic confusion of emotional states (Carbon, 2020), and masks cause significant frequency-dependent transmission loss (Porschmann et al., 2020). In one word, efficient communication is jeopardised (Marler & Ditton, 2021). Furthermore, until early 2020, people in most areas of the world were not accustomed to wearing face masks. Consequently, the acceptance of wearing masks was low in Europe at the beginning of the pandemic, which stands in stark contrast to the commonly high usage rates in various Asian communities (see Landoni et al., 2021; MacIntyre & Chughtai, 2015; van der Sande et al., 2008). Research has recently identified some concrete factors related to personality traits that contribute to explaining individual mask-wearing behaviour. Low compliance with wearing face masks is linked, for instance, with lower levels of empathy but with higher levels of callousness, deceitfulness, and risk-taking (Miguel et al., 2021). Some researchers have also identified gender-specific perceptions of mask-wearing: While women perceived face masks as uncomfortable more often, men felt that face masks restricted their feeling of independence (Howard, 2021). Early correlative studies during the COVID-19 pandemic have identified further person-associated factors, inter alia age (negatively correlated with mask-wearing), perceived infectibility, and recent illness (both positively correlated with mask-wearing; Makhanova & Shepherd, 2020; Shook et al., 2020). Although the summarised studies and results contribute to understanding negative reactions towards mask-wearing, that is only half the story. One essential factor that has not been brought into focus yet relates to the (descriptive) social norm. Many people in the West report the feeling that one may look strange or be judged as being strange by others when wearing a face mask (Friedman, 2020)—at least, this was the case when the current study was performed. The first wave of the COVID-19 pandemic was rising in Germany in April 2020 (with still less than 5,000 deaths overall; Robert Koch Institute, 2020). Feeling “strange” (or “normal,” on the other hand) is closely linked to descriptive social norms that are present in a given social environment. This implies that the mere frequency of mask wearers in society might be an essential factor in moving individuals to wear face masks. This is a paradigmatic example for the importance of understanding the psychology of pandemics (Taylor, 2019). A psychological perspective allows the assessment of why people do or do not do certain things, which is the prerequisite for finding ways to change behaviour. In the case of mask-wearing, the question is as follows: How can we change the attitude towards and the feelings about wearing masks? One possible psychological answer is via the social norm (see Hesslinger et al., 2017a, 2017b). The present study tested this possibility by confronting participants with pictures that show social groups, each with varying frequencies of persons who wear different kinds of face masks. We assessed whether the different social norms that were thus implicitly communicated affected the participants’ feeling about wearing a face mask themselves.

## The Present Study

The present study aims to understand how our feeling to feel strange when imaging to wear an own face mask is modulated by a group of people with persons wearing more or less face masks (from 0, 1, 2, 6 up to 12 out of 12 people in that group). As we hypothesise that a possible effect of the number of people wearing face masks is related to social norms—here, creating mainly a “descriptive norm” (Legros & Cislaghi, 2020, p.67), we were also interested in how participant’s sex played a role in this respect as sex-related processing of social norms were regularly documented (e.g., Felonneau & Becker, 2008; Trelohan, 2021), also in health-related contexts (Fisher, 2009; Magallares & Morales, 2013). Furthermore, we employed a variety of face mask types to increase the heterogeneity of stimuli. We were also interested, which face mask of the types commonly used in everyday life, does induce the lowest strangeness feeling when imagined to use. Knowledge about feelings with specific face masks might help to provide practical advice for policymakers to increase the acceptance of using face masks in publicity.

## Methods

### Participants

Eighty-eight participants volunteered for the study (*M*_age_ = 28.8 years [15–87 years], *N*_female_ = 62). Based on the comparison of Model #2 and Model #1 which directly tested the effect of the number of masks (see details in the Results section), we calculated the test power post hoc via R package *simr* (Green & MacLeod, 2016). For both models to be compared, we set the intercept to 5.0 and the slopes of the fixed effects to +0.1 for loop masks and –0.1 for simple face masks; for Model #2, we set the slope of the fixed effect to –0.1 for the number of mask wearers in the group of people; random intercept variance was set to 1.0 and residual standard deviation to 2.0. Based on the final number of complete data sets of *N* = 88, let us achieve a post hoc test power of 95.0% (95% confidence interval [CI] [75.2%, 99.9%]). (Note: Initially, the required simple sample size was determined according to test planning for linear model, but this turned out to be obsolete due to the change in the analysis strategy).

### Material

**Figure 1.**
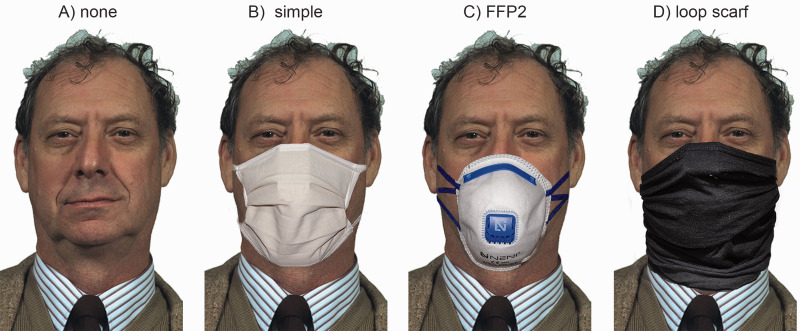
One of the employed faces with different mask conditions: (A) none, (B) simple, (C) FFP2, and (D) loop scarf.

Based on frontal photos of 12 *white* European (previously called “Caucasian”) faces (six female, six male) taken from the color Feret database (Phillips et al., 1998, 2000), we created different versions of displays of these faces. The base version showed all faces without masks at random places in the display making up a social group. For the further displays, we employed different masks which we photographed correctly positioned on an artificial head model: (a) a typical homemade (beige) community mask—in the following called “simple mask,” (b) an FFP2 mask (N95; white), and (c) a black loop scarf (see Figure 1), We cut out the images of the masks via Photoshop to be able to apply them to the different faces of the social group.

**Figure 2.**
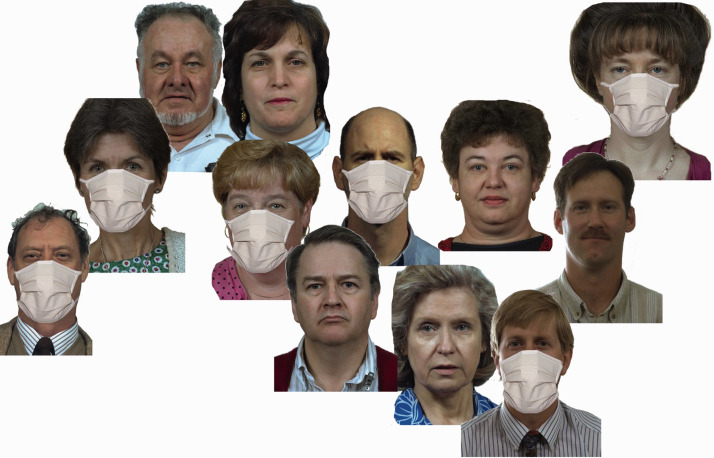
Example display presented, here with six (three female, three male) people wearing simple homemade masks.

For each mask, we generated five different configurations, always consisting of the 12 faces: (a) only one female wearing a mask, (b) only one male wearing a mask, (c) one female and one male wearing masks, (d) three females and three males wearing masks (see Figure 2), and (e) all persons wearing masks. This yielded 1 [base] + 3 [*maskType*: FFP2, loop, simple] × 5 [*nMasks*: number of masks] = 16 versions. The stimulus material can be retrieved from the https://osf.io/gu6xr/.

### Procedure

The experiment, realised via the SoSciSurvey online engine, was conducted between April 20, 2020 (15:47 local time) and April 23, 2020 (16:56 local time). This was before any general legal obligations to wear masks in Germany were in action. Prior to the experimental session, written informed consent was obtained from each participant. All data were collected anonymously. The participants were exposed to all display versions, one after another, with the order of the displays being randomised across participants. Participants were asked to imagine having a face mask on in the social situation signalled by the respective display they viewed (“Imagine: You are wearing a mouth-nose mask yourself and are now facing these persons. How do you feel in such a situation?”). While observing the scene without time pressure (*M*_response time_=20.7 s), they were asked to answer two consecutive questions on a 7-point scale (1= *not strange at all*, 7= *very strange*): (a) *Feel myself*: “While wearing MY mask I am feeling . . . ,” (b) *Feel others*: “The others appear . . . ” (a capitalised “my” was also used in the experimental version). There was no time limit for giving a response. This should allow a full expression of the participants’ imagery for the respective social scene. The participants provided an informed written consent before the experiment started. All procedures were in accordance with the national ethical standards on human experimentation provided by the German Psychological Society (DGPs) and with the Declaration of Helsinki of 1975, as revised in 2008. The study was in full accordance with the ethical guidelines of the University of Bamberg and was approved by an umbrella evaluation for psychophysical testing of the university ethics committee (Ethikrat) on August 18, 2017. Specific ethical approval beyond these means was not sought for the present study because the experimental treatment was not susceptible to trigger particularly negative emotions beyond typical displays of humans with face masks in public life, also taking into account that minors of 15+ years were involved (*n* = 6 consisting of one 15-year-old person with special permission of his parents and five 17-year-old persons). The entire procedure lasted approximately 5–10 minutes.

## Results

### Data Analysis Strategy

The data were processed using the R 4.0.4 (R Core Team, 2021). In addition to the *lme4* package (Bates et al., 2015) to perform linear mixed effects analyses, R packages *lmerTest* (Kuznetsova et al., 2017) and *ggplot2* (Wickham, 2012) were used during the analysis of the data. The entire anonymised data set is available at the Open Science Framework https://osf.io/gu6xr/.

As we were mainly interested in the impact of mask-wearing of others on our two dependent variables, we initially defined a linear mixed model as null model (Model #0) with no fixed effects but only the participants as random factor. We subsequently added effects of interest and tested these models against the respective models without the effects in question via likelihood ratio tests. Each model’s residuals were visually inspected to exclude models deviating from homoscedasticity or normality. Table 1 shows the subsequent testing of the models towards best fitting. Table 2 depicts the estimates of finally selected models for the dependent variables “strangeness regarding myself” (strangeness-myself) and “strangeness regarding others” (strangeness-others).

**Table 2.** Final Models for the Dependent Variables “Feel Strange (Myself)” and “Feel Strange (Others)”.

	Strangeness-myself (final) Model #4			Strangeness-others (final) Model #4		
Predictors	Estimates	p	df	Estimates	p	df
(Intercept)	5.63***	**<.001**	112.40	2.07***	**<.001**	140.11
*maskType*						
Simp	*Reference*			*Reference*		
FFP2	0.13*	**0.010**	1403.00	0.43***	**<.001**	1403.00
Loop	0.26***	**<.001**	1403.00	0.54***	**<.001**	1403.00
*nMasks*						
None	*Reference*			*Reference*		
1	–0.92***	**<.001**	1489.99	0.85***	**<.001**	1489.14
2	–1.30***	**<.001**	1264.77	1.15***	**<.001**	1311.93
6	–2.02***	**<.001**	193.48	1.47***	**<.001**	206.36
All	–2.61***	**<.001**	95.54	1.43***	**<.001**	96.44
*sSex*						
Female	*Reference*			*Reference*		
Male	–0.60*	**0.043**	86.00	0.26	0.290	86.00
ICC	0.73			0.59		
*N*	88 _Subj_			88 _Subj_		
Observations	1,584			1,584		
Marginal *R*^2^/Conditional *R*^2^	0.232/0.792			0.091/0.627		
AIC	4497.470			5351.160		
log-likelihood	–2236.735			–2663.580		

*Note*. For both dependent variables, Model #4 was independently selected due to best respective fits. AIC = Akaike information criterion; ICC = intraclass correlation coefficient. Bold p values indicate significant results.

*p < .05. ***p < .001.

The null model (Model #0) employed the participants (*Subj*) as random (intercept) factor only. Model #1 added *maskType* as fixed factor. For Model #2, we added *nMasks* as fixed ordered factor; for Model #3, we furthermore added the participants’ sex (sSex) as fixed factor. Model #4 further extended the model by adding *nMasks* as random (slope) factor. For unordered as well as ordered factors, treatment contrasts were used.

### Dependent Variable: Strangeness-Myself

Regarding the first dependent variable on the feeling strange about one’s own wearing a mask (*strangeness-myself*), we were able to identify Model #4 as the best fitting. This model explained 79.2% of the variance (see Table 2). For this final model (see Table 1 for details about the selection of the model), we revealed *maskType* as a significant fixed effect, with *FFP2* masks and *loop* scarfs showing higher *strangeness-myself* ratings than *simple* face masks. The estimate for loop scarfs was doubled compared to FFP2 masks. Regarding the number of masks available in the social group of people (*nMasks*), we uncovered effects for all comparisons of numbers against the “none” condition, where none of the people wore a mask. A deeper look revealed that the decrease of feeling strange about one’s own wearing a mask was closely related to the number of mask wearers in the social group. All estimates were much larger in absolute numbers than for the factor *maskType*, illustrating a relative large (negative) influence of *nMasks* on *strangeness-myself* ratings. Furthermore, male participants expressed a lower feeling strange about one’s own wearing a mask.

**Table 1.** Comparison of Models for Both Dependent Variables.

Model	*Npar*		AIC	–2LL	*df*	χ^2^	*p*
Dependent variable: *strangeness-myself*							
#0: 1+(1|Subj)		3	5679.7	2836.8			
#1: 1+maskType+(1|Subj)		5	5674.1	–2832.1	2	9.6	.0083*
#2: 1+maskType+nMasks+(1|Subj)		9	4884.7	–2433.4	4	797.3	<.0001***
#3: 1+maskType+nMasks+sSex+(1|Subj)		10	4882.1	–2431.0	1	4.7	.0308*
#4: 1+maskType+nMasks+sSex+(1+nMasks|Subj)		12	4473.1	–2224.5	2	413.0	<.0001***
Dependent variable: *strangeness-others*							
#0: 1+(1|Subj)		3	5921.4	–2957.7			
#1: 1+maskType+(1|Subj)		5	5885.2	–2937.6	2	40.2	<.0001***
#2: 1+maskType+nMasks+(1|Subj)		9	5698.4	–2840.2	4	194.8	<.0001***
#3: 1+maskType+nMasks+sSex+(1|Subj)		10	5699.0	–2839.5	1	1.4	.2409 *n.s.*
#4: 1+maskType+nMasks+sSex+(1+nMasks|Subj)		12	5329.5	–2652.8	2	373.5	<.0001***

*Note*. Npar = number of model’s parameters; AIC = Akaike information criterion, an estimator of prediction error; –2LL = likelihood ratio; *df*, *p* = degrees of freedom and *p* value of the regarding χ^2^ test (comparing the present model with the preceding one, e.g., the columns for Model #3 indicate the comparison between Model #3 and Model #2).

### Dependent Variable: Strangeness-Others

Regarding the second dependent variable on the feeling strange about others wearing a mask (*strangeness-others*), we were able to identify (again) Model #4 as the best fitting. This model explained 62.7% of the variance (see Table 2). For this final model (see Table 1 for details about the selection of the model), we revealed *maskType* as a significant fixed effect, with *FFP2* masks and *loop* scarfs showing higher *strangeness-others* ratings than *simple* face masks. The estimate for loop scarfs was just a bit larger compared to FFP2 masks. Regarding the number of masks available in the social group of people (*nMasks*), we uncovered effects for all comparisons of numbers (1, 2, 6, 12 = *all*) against the “none” condition, where none of the people wore a mask. All estimates were much larger than for the factor *maskType*, illustrating a relative large (positive) influence of *nMasks* on *strangeness-others* ratings.

### Overall View: Strangeness-Myself Versus Strangeness-Others

**Figure 3.**
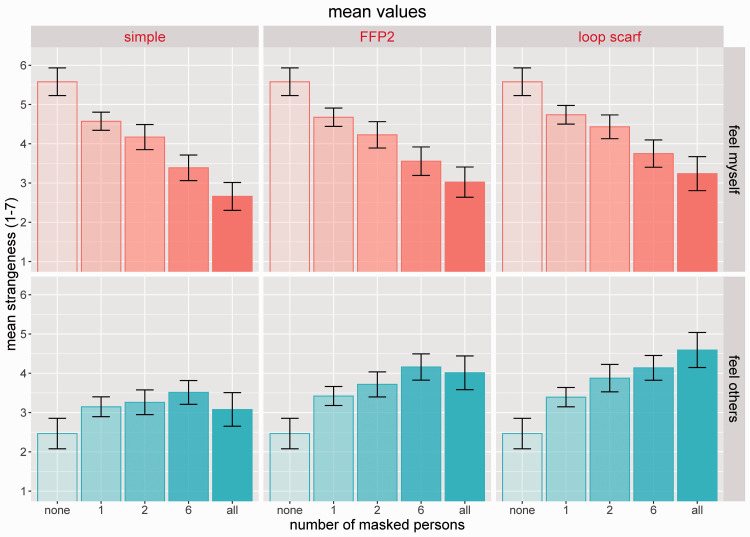
Mean evaluations of strangeness for different displays. Top row: evaluations of participants feeling strange about themselves (“feel myself”) while watching the displays. Bottom row: evaluations of others appearing strange (“feel others”). Error bars indicate confidence intervals (95% CI) based on adjusted values for taking within-subjects variances into account (Morey, 2008).

As shown in Figure 3, we uncovered a clear decrease of feeling strange about one’s own wearing a mask (*strangeness-myself*) with increasing numbers of masks (*nMasks*) worn by the people depicted in the group. Meanwhile, the participants evaluated the appearance of the persons shown in the social scene as being increasingly strange with increasing numbers of mask wearers in the group (*strangeness-others)*. Both effects were found to be significant when tested with linear mixed models against a null model without taking the number of mask wearers into account (*p*’s<.0001). Based on the final models (#4), the estimates in absolute numbers for the factor *nMasks* were much larger for *strangeness-myself* than for *strangeness-others*. This means that the clear decrease in feeling strange about oneself when more persons wore face masks in the group of people was not only accompanied by a reversed effect of feeling strangeness (oddness) of the group of viewed people but was also more pronounced.

## Discussion

Wearing face masks in times of COVID-19 is one of the essential keystones for effectively decreasing the rate of new infections and for mitigating the negative consequences for individuals as well as for society. Wearing masks does not belong to natural human’s habits and is still not easily acceptable for many people (Wong, 2020) and has been emerged as a political issue (Rabinovitch-Fox, 2020)—many people just feel strange when wearing masks (Robb, 2021) and therefore will not follow recommendations to put on masks in public. Here, we tested how the mere exposure to people in the social environment who do or do not wear masks can dramatically change the feeling of strangeness when wearing a mask oneself.

It is of particular interest that the number of mask wearers had dissociative effects on both dependent variables employed in the present study: The participants experienced the idea of wearing masks themselves as less and less strange when more people in the shown social group wore face masks as well. At the same time, however, they kept perceiving the other mask wearers in the displayed social group as strange, especially when they wore loop scarfs, in this case, black, loop scarfs. We suggest that this dissociation of effects is the outcome of two different mechanisms that are at work here: A more perceptual one and a more cognitive (normative) one. To illustrate this, we would like to give an example: Imagine you are invited by a good friend who grew up in Venice to visit his/her beautiful hometown to which you have never been. You travel to Venice, and upon arriving there in a small taxi boat, you realise that the world-famous Carnival of Venice is well underway. People all around you, including your friend who is welcoming you at the landing stage, are wearing the typical, highly elaborate masks. You were not prepared for the festival, so you do not have a mask. You will, most probably, experience the following: The people around you will appear somewhat strange to you—this mainly *perceptual* effect is based on an insufficient familiarity with the specific disguise. Furthermore, with such masks on, we cannot rely anymore on typical processes which we effortlessly use in normal, everyday life without any masks, for example, reading the emotional state (Carbon, 2020) and further mental states (Schmidtmann et al., 2020) of others by merely processing the holistic facial information. Yet, you will probably feel less strange about yourself as soon as you put on a mask as well—this effect traces back to the descriptive social norm that is established by the outward appearance (the shared dress code) of the majority of people around you in this specific situation. This effect of taking social norms into account is a *cognitively* based effect. It is important to understand this perceptuo-cognitive dissociation because it is not limited to wearing masks: We often adopt descriptive social norms that are signalled by the empirical conditions of present situations, and we try to behave like the others around us, but this does not necessarily mean that we like or would principally endorse this behaviour as well. In the present experiment, the perception of others as being strange was particularly strong for *loop* scarfs and *FFP2* masks. The *loop* scarfs resemble so-called bandanas—may be because of negative connotations triggered by the resemblance with the cliché masking of bank robbers in movies or cartoons. The *FFP2* masks, at least at the early phase of the pandemic when this study was conducted, were obviously also seen as being strange—but probably due to another phenomenon: Most people were unfamiliar with this kind of mask which should have fundamentally changed meanwhile due to the everyday usage of such masks.

So, which masks seem to be optimal for everyday usage? From a physical (Verma et al., 2020), mathematical (Mittal et al., 2020) as well as a medical (Chu et al., 2020) perspective, there are clear answers to this question: The mask should be capable of filtering a maximum of airborne particles, so the certified face masks with FFP2 (N95; filtering at least 95% of airborne particles, if they show a diameter of at least 100 nm; O'Dowd et al., 2020) and FFP3 (N99; 99%) filtering levels seem to be the best (O'Dowd et al., 2020). From a psychological perspective, the answer might differ. In the present study, we observed least perceived strangeness when observing other people wearing less intrusive masks, concretely simple self-made masks, while *loop* scarfs and *FFP2* masks showed higher levels of perceived strangeness in this respect. Meanwhile, participants did not feel particularly strange themselves, actually even a bit less strange than the others shown as a social group. Such simple face masks offer a series of other advantages: First, they are relatively easy and comfortable to use (Yao et al., 2019), they can be easily and privately produced by simple means, and they are cheap enough to equip many people around the globe in high quantity and fresh quality. Second, as the suggestions for wearing masks for private persons refer to the protection of others and because there is no clear evidence of a difference in protecting others between simple masks and FFP2/N95 masks (Jefferson et al., 2020), simple masks prevent a shortage of professional medical masks that should be primarily reserved for medical workers. Third, in our study, the simple masks showed the highest acceptance rate in terms of feeling least odd when imaging wearing such a mask. This is an important precondition to face masks actually being worn in different situations and over a longer period (see MacIntyre et al., 2009; MacIntyre & Chughtai, 2015), especially by non-medical workers (Matusiak et al., 2020). Furthermore, they do not emit large amounts of microplastic fibres as one-way masks might do (Fadare & Okoffo, 2020). Of course, such general ideas should be adjusted for specific contexts and fields of applications, for example, it was shown that wearing masks has race-specific effects on other perceivers (Christiani et al., in press). This last point also calls for extensions of such studies as we only tested a relatively narrow sample employing *White* European faces only wearing three different types of face masks that were popular and available in April 2020. For instance, there are reports and societally meaningful discussions on interactive effects between wearing specific masks and ethnic background and the morphological group of the wearer, for example, black bandanas worn by people of colour which triggered racial stereotypes (Ray, 2020). This should be systematically analysed to understand and to counteract against such mechanisms.

In general, our results will also assist policymakers in predicting the future acceptance of wearing masks in which generally more people comply with these new hygienic practices, following role models wearing masks and propagating them instead of denying and problematising them (Hornsey et al., 2020).

## Note

1 The term *mask* will be used within this article to refer to typical face masks used by non-professionals, including improvised masks, for example, loop scarfs, dust, and surgical masks—such protective items are now frequently called *community masks*.
